# Health outcomes, education, healthcare delivery and quality – 3040. Occurrence and effects of nasal polyps in patients with bronchial asthma and/or allergic rhinitis

**DOI:** 10.1186/1939-4551-6-S1-P213

**Published:** 2013-04-23

**Authors:** Swati Behera

**Affiliations:** 1 Pulmonary Medicine, Vallabhbhai Patel Chest Institute, New Delhi, India

## Background

The occurrence of nasal polyps (NP) in bronchial asthma (BA) and/or allergic rhinitis (AR) remains largely undiagnosed and adversely affects quality of life (QoL).

## Methods

The study comprised 220 consecutive patients (males/females, 123/97), 15 to 60 years with BA and/or AR enrolled from outpatients department of VP Chest Institute, University of Delhi. BA/AR was diagnosed according to GINA/ARIA guidelines respectively. Group1 comprised 35 patients with BA, group2, 43 patients with AR and group3, 142 patients with both diseases. CT-PNS, done in all patients, assessed CRS/NP and was scored with Lund Mackey Score (LMS). To assess the effect of NP, asthmatics responded to asthma QoL questionnaire (MiniAQLQ), and the 15-17 years to MiniPAQLQ. Rhinitics responded to rhinoconjunctivitis QoL questionnaire (RQLQ) and the 15-17 years to AdolRQLQ. Patients with nasal symptoms responded to Sinonasal Outcome Test 22 (SNOT 22) and Visual Analogue Scale (VAS).

## Results

Of the 220 patients, 190(86.4%) had CRS. Of these, 138(72.6%) had NP. CRS was seen in 26/35(74.3%) patients in group1, 38/43(88.4%), in group2 and 126/142(88.7%), in group3. NP was seen in 18/26(69.2%), 30/38(78.9%) and 90/126(71.4%) in groups 1, 2 and 3 respectively. The presence of NP increased mean LMS score from 5 in CRS to 8(*P*=0.005) in CRS/NP patients. In group2, occurrence of NP increased mean Global VAS score from 5 to 7(*P*=0.029), SNOT 22 scores from 43 to 44(*P*=0.029) and in RQLQ scores, activities score rose from 1.7 to 3.1(*P*=0.007), nasal symptom score from 3.9 to 4.1(*P*=0.033), non hayfever symptom score from 2.3 to 3.5(*P*=0.045) and RQLQ overall scores from 2.4 to 3.1(*P*=0.023). In group3, NP increased mean SNOT 22 scores from 39 to 42(*P*= 0.002), RQLQ nasal symptom score from 4.2 to 4.4(*P*=*0.001*) and RQLQ troubled sleep score from 1.7 to 1.9(*P*=0.021). There were no significant differences in group1. In all three groups, presence of NP increased mean RQLQ activities score from 3 to 3.3(*P*=0.033) and RQLQ troubled sleep score from 1.7 to 2(*P*=0.010).

## Conclusions

NP was seen in nearly two-third (62.7%) of patients with BA and/or AR. QoL was maximally impaired when AR was complicated with NP followed by patients with both diseases.

